# Penetrating aortic ulcer in the aortic arch repaired by a novel double inner-branched stent-graft

**DOI:** 10.1093/ehjcr/ytae003

**Published:** 2024-01-04

**Authors:** Xiaolang Jiang, Zhihui Dong, Weiguo Fu

**Affiliations:** Department of Vascular Surgery, Institute of Vascular Surgery, Zhongshan Hospital, Fudan University, 180 Fenglin Road, Shanghai 200032, China; National Clinical Research Center for Interventional Medicine, 180 Fenglin Road, Shanghai 200032, China; Department of Vascular Surgery, Institute of Vascular Surgery, Zhongshan Hospital, Fudan University, 180 Fenglin Road, Shanghai 200032, China; National Clinical Research Center for Interventional Medicine, 180 Fenglin Road, Shanghai 200032, China; Department of Vascular Surgery, Institute of Vascular Surgery, Zhongshan Hospital, Fudan University, 180 Fenglin Road, Shanghai 200032, China; National Clinical Research Center for Interventional Medicine, 180 Fenglin Road, Shanghai 200032, China

A 76-year-old male patient complained of recurrent chest pain for 3 months. The computed tomography angiography (CTA) revealed a penetrating aortic ulcer (PAU) in the aortic arch (*Panel A*). The patient had hypertension, diabetes mellitus, and hyperlipidaemia, and he was identified at high risk for open repair after the multidisciplinary consultation. We aimed to repair the lesion with a novel device. Preoperative digital subtraction angiography (DSA) also confirmed the diagnosis (*Panel B*). The left common carotid artery (LCCA)–left subclavian artery (LSA) bypass was firstly performed, and the ostium of LSA was ligated to prevent endoleak. Then a novel modular double inner-branched stent-graft (Hangzhou Endonom Medtech Co., Ltd, Hangzhou, China; *Panel C*; Supplementary material online, *Figure S1*) was applied to repair the PAU and reconstruct the innominate artery (IA) and LCCA (*Panels D–G*; Supplementary material online, *Video S1*). Technical success was achieved (*Panel H*). At the 6-month follow-up, the PAU was no longer visualized and all stent-grafts were patent without migration by CTA (*Panel I*). The patient felt no more chest pain.

Although open aortic repair was the first-line treatment for aortic arch pathologies, it also carries significant morbidity and mortality, especially in high-risk patients. This novel device is undergoing clinical trials in our nation, and it has several anatomical criteria, including (i) ascending aorta length ≥50 mm (from the sinotubular junction to the proximal margin of the IA), (ii) ascending aorta diameter ≥24 and ≤48 mm, (iii) proximal anchoring zone length ≥30 mm, (iv) IA diameter ≤24 and ≥7 mm and length ≥20 mm, and (v) LCCA or LSA diameter ≤24 and ≥7 mm and length ≥ 20 mm. This case showed safety and efficacy of this novel inner double-branched stent-graft in the treatment of aortic arch pathologies. It could avoid deep hypothermic circulatory arrest and reduce cerebral ischaemia time, which may emerge as a promising solution to aortic arch pathologies in high-risk patients.

**Figure:**
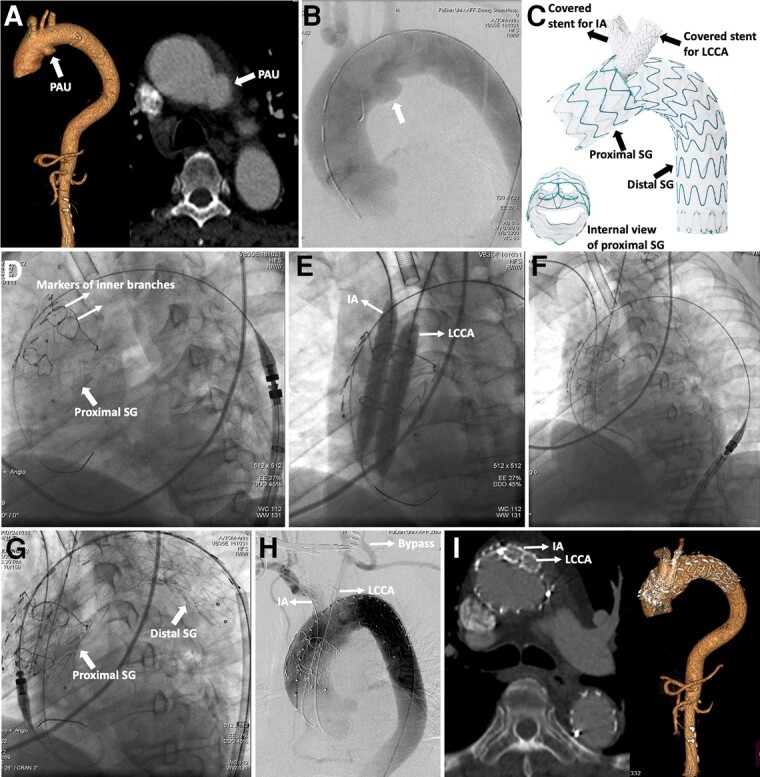
(*A*) Computed tomography angiography confirmed penetrating aortic ulcer in the aortic arch. (*B*) Digital subtraction angiography confirmed the diagnosis. (*C*) Design of the novel double inner-branched device. (*D*) Deployment of the stent-graft in the ascending aorta. (*E*) Confirmation of two inner branches with balloons. (*F*) Deployment of bridged covered stents in the innominate artery and left common carotid artery. (*G*) Deployment of the distal stent-graft. (*H*) Final angiography showed procedural success. (*I*) Computed tomography angiography was followed up for 6 months. PAU, penetrating aortic ulcer; IA, innominate artery; LCCA, left common carotid artery; SG, stent-graft.

## Supplementary Material

ytae003_Supplementary_Data

## Data Availability

Data available on request from the authors.

## Supplementary material

**Consent:** The authors confirm that written consent for submission and publication of this case report, including images and associated text, has been obtained from the patient in line with Committee on Publication Ethics guidance.

**Funding:** This work was supported by the National Natural Science Foundation of China (grant numbers 82270507 and 81770508).

