# Supplementary figures and images for: Penetrating aortic ulcer in the aortic arch repaired by a novel double inner-branched stent-graft
Source: Eur Heart J Case Rep. 2024 Jan 4;8(1):ytae003. doi: 10.1093/ehjcr/ytae003 (PMC10787361; doi:10.1093/ehjcr/ytae003)

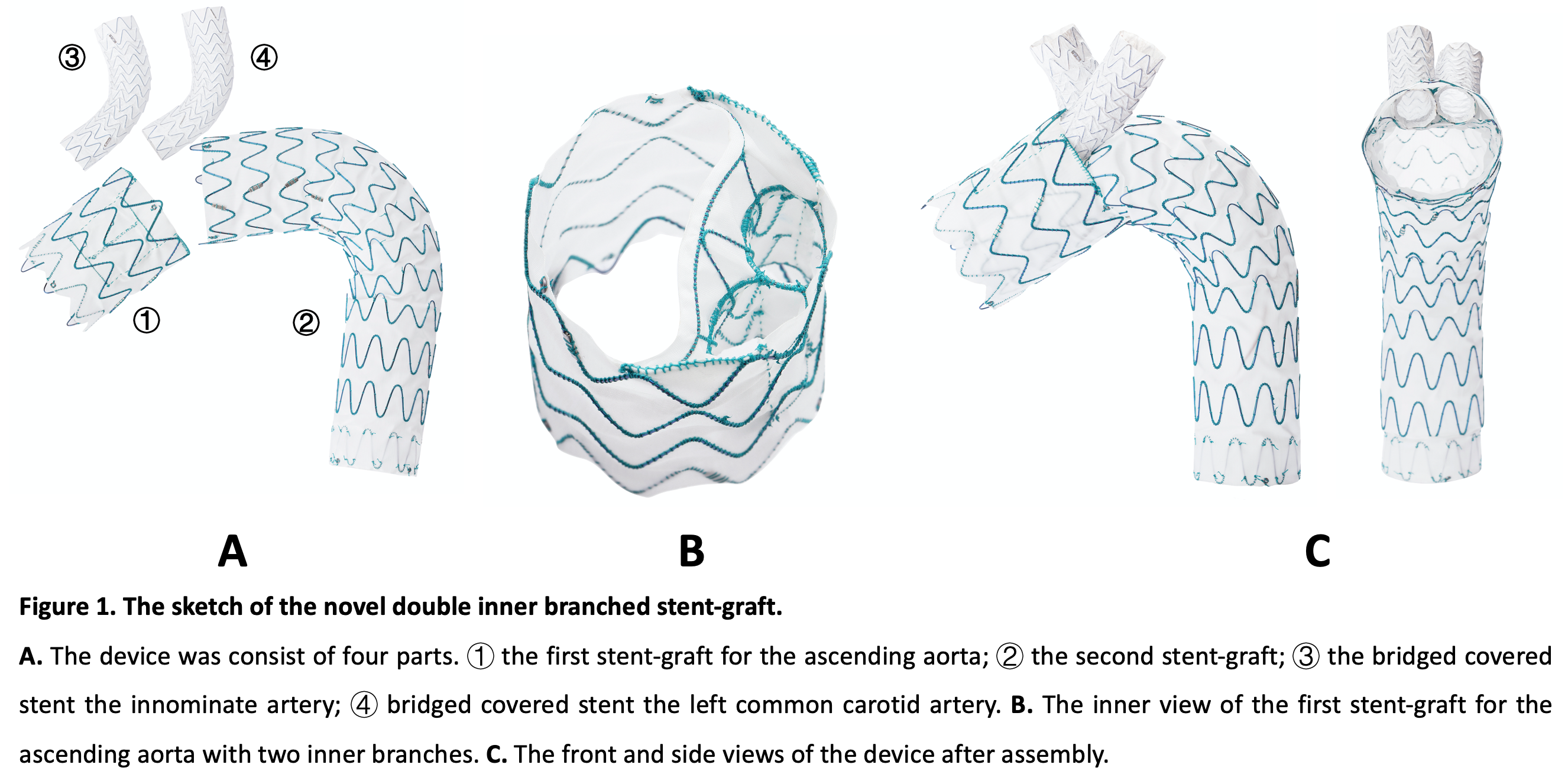

Supplement: ytae003_Supplementary_Data [file ytae003_supplementary_data.zip › Supplementary Figure 1.tif]
